# Supplementary material for: Fast and scalable inference of multi-sample cancer lineages
Source: Genome Biol. 2015 May 6;16(1):91. doi: 10.1186/s13059-015-0647-8 (PMC4501097; doi:10.1186/s13059-015-0647-8)
Supplement: Additional file 1 — Evaluation of Phylosub. Performance evaluation of the Phylosub [29] program on the ccRCC dataset. [file 13059_2015_647_MOESM1_ESM.zip › Phylosub-evaluation/Evaluation.pdf]

# Evaluation of PhyloSub

We ran PhyloSub [29] on the Gerlinger *et al* dataset [21] using default parameters. For each patient in the dataset we compared the top tree structures (by default, three) with the published tree. It is hard to find similarities between the topology of reported trees and the published trees. Therefore, we analyzed the performance of PhyloSub in finding the correct clusters of mutations, which is a preliminary requirement for building the cancer progression pathway. Gerlinger et. al have categorized mutation groups as shared, heterogeneous (partially shared), and private. Generally speaking, we observed that shared mutations were clustered correctly but partially shared mutations were often clustered all together as one group, which violates their presence pattern. As an example, PhyloSub reported a tree for patient EV003 with three mutation groups (see Figure 1 below). The trunk branch (colored blue) includes all shared mutations as well as one private and two partially shared mutations. Second branch (colored green) contains several partially shared mutations and the rest of private mutations with very different presence patterns, and the third branch (colored red) contains three partially shared mutations. Case EV003 has a heterogenous group with 8 mutations shared by R9 and R6 (LONRF2, RHOB, BHLHE40, CMYA5, NOD1, GCC1, SLC5A12, YLPM1), whereas PhyloSub puts two of them in the trunk branch, four in the second, and two in the third brach. Note that, as discussed in the Results section, for EV003 LICHeE was able to find all mutation groups and build the lineage matching the reported tree in Gerlinger et. al dataset. For other cases PhyloSub showed similar performance (see the supplementary results in the folder). We also observed that PhyloSub does not deal with private mutations properly. Often private mutations are either clustered into singleton groups or are distributed into shared or partially shared groups. For example, for six out of eight patients in at least one of the top-trees there are private mutations clustered with shared mutations and located in the trunk branch of the corresponding tree. Note that PhyloSub shows acceptable performance in analyzing samples with very few mutations and simple (chain) topology. However, when tested on more complex multi-sample cancer datasets, the performance of LICHeE is far more superior.

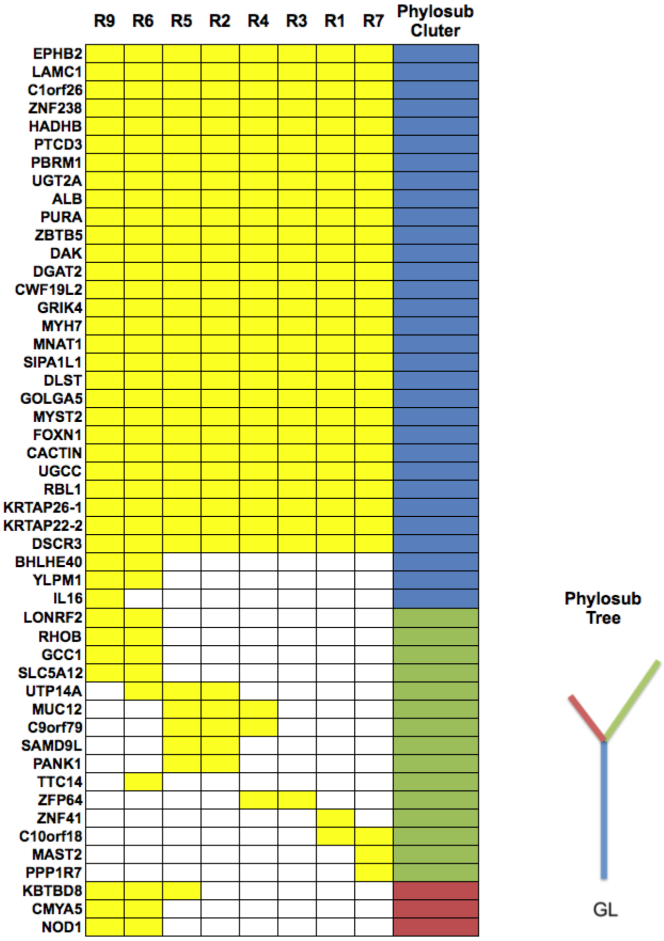


Figure 1: PhyloSub results for patient EV003.
