# Supplementary material for: Fast and scalable inference of multi-sample cancer lineages
Source: Genome Biol. 2015 May 6;16(1):91. doi: 10.1186/s13059-015-0647-8 (PMC4501097; doi:10.1186/s13059-015-0647-8)
Supplement: Additional file 2 — Experimental details of the ccRCC, HGSC, and xenoengraftment comparison. It includes a description of the dataset and LICHeE parameter settings. [file 13059_2015_647_MOESM2_ESM.pdf]

Experimental Details of the ccRCC, HGSC, and Xenoengraftment Comparison: Dataset Description and Parameter Settings

ccRCC. The clear cell renal cell carcinoma (ccRCC) study by Gerlinger et. al [21] validated 602 nonsynonymous nucleotide substitutions and indels from multiple samples of 8 individuals using ultra-deep amplicon sequencing with an average depth of >400x. The analysis was performed on 587 out of these mutations guaranteeing a minimum coverage higher than 100x. Based on the expected sequencing platform error rate, mutations were called in a sample if their VAF was $\geq$ 0.5% for substitutions and 1% for indels. In accordance with the study, we set the cutoffs to $T_{present} = T_{absent} = 0.005$ for calling SSNVs across samples for all patients except RMH004 and required at least two mutations as evidence of a node in the tree. For RMH004 we set $T_{present} = T_{absent} = 0.01$ to allow editing, observing that some mutations with the frequencies slightly higher that 0.5% were considered absent in some samples of the study.

HGSC. In the high-grade ovarian cancer (HGSC) dataset from the study by Bashashati et. al. [27], 19 tumor samples from six patients were used to validate 340 somatic mutations using deep amplicon sequencing (with a median coverage of $>$ 5000x). When running LICHeE, we used the hard thresholds of $T_{absent} = 0.005$ and $T_{present} = 0.01$ for all the patients except Case5, where we used $T_{absent} = 0.01$ and $T_{present} = 0.04$ due to a higher level of noise in the data, and required at least three mutations as evidence of a node in the tree. For Case5 we also removed the samples g and h from consideration since they had multiple inconclusive validation results as stated in the manuscript [27]. The explicit command-line settings used for each patient are listed in the project repository `https://github.com/viq854/lichee`, see file LICHeE/data/README.

Xenoengraftment. The study by Eirew et. al. [32] used deep-genome and single-cell sequencing to evaluate the clonal dynamics of xenoengraftment of breast cancer tissue into immunodeficient mice. Single-cell analysis was done on passages SA501 (samples X1, X2, and X4) and SA494 (samples T and X4). When running LICHeE on both passages, we used the hard thresholds of $T_{absent} = 0.03$ and $T_{present} = 0.05$ (the value 0.05 was indicated in the study's supplementary materials); a minimum cluster size of 6 for non-private mutations and 5 for private mutations; and a maximum cluster collapse distance of 0.085.
